# Quality by Design Approach in Liposomal Formulations: Robust Product Development

**DOI:** 10.3390/molecules28010010

**Published:** 2022-12-20

**Authors:** Walhan Alshaer, Hamdi Nsairat, Zainab Lafi, Omar M. Hourani, Abdulfattah Al-Kadash, Ezaldeen Esawi, Alaaldin M. Alkilany

**Affiliations:** 1 Cell Therapy Center, The University of Jordan, Amman 11942, Jordan; 2 Pharmacological and Diagnostic Research Center, Faculty of Pharmacy, Al-Ahliyya Amman University, Amman 19328, Jordan; 3 Department of Pharmaceutics and Pharmaceutical Technology, School of Pharmacy, The University of Jordan, Amman 11942, Jordan; 4 College of Pharmacy, QU Health, Qatar University, Doha 2713, Qatar

**Keywords:** drug delivery, nanomedicine, liposomes, quality by Design (QbD), nano-pharmaceuticals, pharmaceutical industry

## Abstract

Nanomedicine is an emerging field with continuous growth and differentiation. Liposomal formulations are a major platform in nanomedicine, with more than fifteen FDA-approved liposomal products in the market. However, as is the case for other types of nanoparticle-based delivery systems, liposomal formulations and manufacturing is intrinsically complex and associated with a set of dependent and independent variables, rendering experiential optimization a tedious process in general. Quality by design (QbD) is a powerful approach that can be applied in such complex systems to facilitate product development and ensure reproducible manufacturing processes, which are an essential pre-requisite for efficient and safe therapeutics. Input variables (related to materials, processes and experiment design) and the quality attributes for the final liposomal product should follow a systematic and planned experimental design to identify critical variables and optimal formulations/processes, where these elements are subjected to risk assessment. This review discusses the current practices that employ QbD in developing liposomal-based nano-pharmaceuticals.

## 1. Introduction

Nanomedicine and nanoparticle-based therapeutics are gaining increasing interest in both academia and industry. Currently, there are many FDA-approved nanomedicine products with proven clinical outcomes [1]. Liposomes are spherical vesicles of a continuous three-dimensional phospholipids bilayer wrapping an aqueous core [2]. Liposomes have been used to deliver a wide range of therapeutics [3]. For example, liposomes have been successfully loaded with the anticancer agent, doxorubicin, and showed enhanced therapeutic efficacy and decreased unwanted side effects [4]. Moreover, they have been widely investigated as carriers of nucleic acid-based therapies, such as siRNA [5] and DNA, enabling enhanced penetration in targeted cells and protecting drugs from degradation [5]. Liposomes were one of the first nanotechnology-platforms that entered the market early in 1995 and is still one of the major nano-platforms [1]. It is worth mentioning that the first FDA-approved mRNA vaccine for COVID-19 was approved in 2020 utilizes lipidic/liposomal nanocarriers as a delivery system [6]. Despite the outstanding properties of liposomes, the complexity in their formulations, product development and manufacturing are clearly challenging. The explanation of increased complexity in the case of nano-formulations/nanomanufacturing is associated with the unique physics and chemistry at the nanoscale and thus a higher number of variables needed to be understood and optimized [7]. Lack of this understanding and optimization is the reason behind the common sensitivity and poor reproducibility in nano-preparations and manufacturing. For these systems, an experimental approach that facilitates the identification of critical parameters and help in understanding their contributions to the characteristics/quality of the final product is certainly beneficial. For this purpose, the quality by design (QbD) has been proposed and recommended by various industries and regulatory agencies [8,9]. QbD starts by identifying the quality target product profile (QTPP), which is a summary of the quality attributes (QA) of the final product to ensure its efficacy and safety. QA is dependent on critical attributes related to the material attributes (CMA) and process parameters (CPP). QbD follows by identifying and optimizing CMA and CPP and setting their target specifications to ensure the QA and ultimately QTPP for the final product [9,10,11]. Proper experimental design is used to link CMA and CPP to QA [8,12], which then facilitate the establishment of targeted specifications for materials, processes and the final product. Moreover, QbD enables the evaluation of the effect of more than one factor at a time on the QTPP. Additionally, risk assessments are used to prioritize QA [13]. Considering the potent liposomal-based drug products in clinical use and the diverse clinical and pre-clinical applications, there is an unmet need for strategic and systematic development of liposomes as potent drug delivery systems that enable better therapeutic efficacy of the loaded therapies. Although applying QbD liposomal drug delivery systems development have been described in several research, there is more and more need to understand and describe current advances in using QbD in liposomal formulation developments to guarantee liposomal-based drug delivery systems with higher therapeutic outcomes and possible industrial development. Therefore, this review highlights the main strategic points of developing liposomes according to the QbD to reduce the obstacles of using such vehicles in clinical applications in the future.

## 2. Quality by Design (QbD)

### 2.1. QbD in Pharmaceutical Products

**Figure 1:**
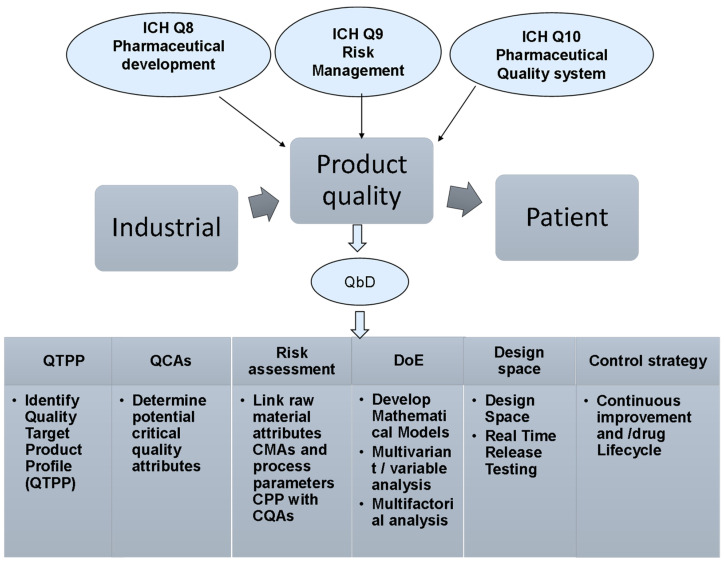
The pharmaceutical development guidelines suggested by ICH, US FDA and EMA to outline the QbD key elements to ensure the consistency of high-quality pharmaceutical products.

The production of quality pharmaceutical products is the major goal of pharmaceutical industry [14]. The quality of the pharmaceutical products covers all aspects that may have an impact on the prescribed products which will consequently affect the health of the patients. Previously, the quality by testing method (QbT) was the common method to ensure quality of the manufactured products. QbT is based on an in-process testing of input materials, intermediates and the final product [15]. However, the pharmaceutical quality sectors call for an alternative practice that can ensure the quality before manufacturing in addition to maintaining the required quality control testing suggested by QbT. To this end, the current pharmaceutical industry and regulation firms switch toward what is now known as the QbD, which ensures that pharmaceutical products will be developed and manufactured as per pre-defined quality attributes, thus QbD is expected to minimize intensive testing during or after manufacturing as well as improve reproducibility, manufacturability, efficacy and safety [16]. Therefore, QbD can be defined as a prospective approach to improve product quality [17]. ICH, US FDA and EMA have specified thoroughly the outlines of the QbD key elements to ensure the consistency of high-quality pharmaceutical products (Figure 1), reflecting a continuous interest in QbD implementation by various international regulatory bodies [16,18].

### 2.2. Tools and Key Elements of QbD

**Figure 2:**
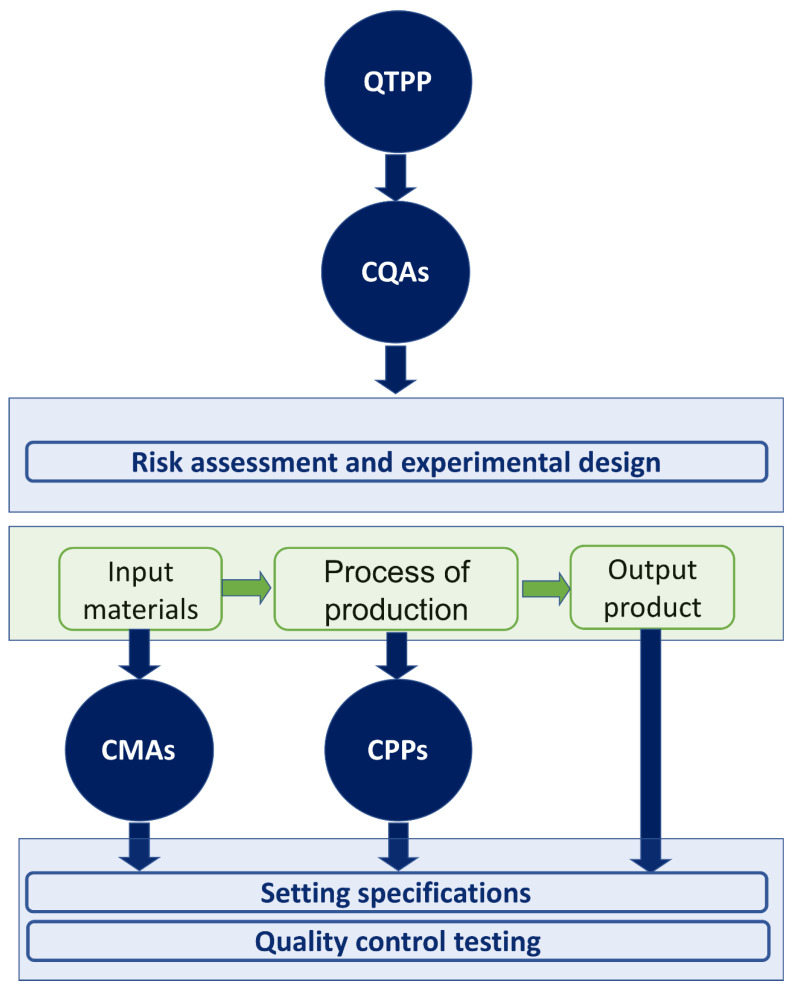
QbD roadmap, and QTPP and CQAs key elements.

Generally, there are four key elements of the QbD: (i) the quality target product profile (QTPP), (ii) the critical quality attributes (CQAs), (iii) the critical material attributes (CMAs) and (iv) the critical process parameters (CPPs) [12,19,20]. All of these elements are collaborating in a step-by-step approach to draw the framework of the QbD strategy. The recruitment of these key elements in the QbD method needs well-defined experimental design combined to proper statistical analysis (Figure 2) [16,21].

ICH guidelines define QTPP as “a prospective summary of the quality characteristics of a drug product that ideally will be achieved to ensure the desired quality, taking into account safety and efficiency of the drug product” [16,18]. To identify QTPPs and define the desired performance of the product, the manufacturer should consider complex variables, such as drug pharmacokinetic parameters, product stability, sterility and drug release [22]. The critical quality attributes (CQAs) were defined by ICH Q8 guideline as “physical, chemical, biological, or microbiological property or characteristic that should be within an appropriate limit, range, or distribution to ensure the desired product quality.” In light of this definition, the CQAs are derived from the QTPP, regulatory requirements, or available literature knowledge. Thus, the critical Quality attribute (CQA) of the drug product and its QTPP is the basis of its dosage form, excipient and manufacturing process selection [23].

The critical process parameters (CPPs) are the process-related parameters that significantly affect the QTPP [16]. The identification of CPPs, an in-depth understanding of the developed standards/specifications, and linking CMAs and CPPs to CQAs are crucial to ensure quality products [24]. Furthermore, both critical material attributes (CMAs) and critical process parameters (CPPs) are generally defined as “A material or process whose variability has an impact on a critical quality attribute and should be monitored or controlled to ensure the desired drug product quality” [23]. It is worth mentioning that CMAs are for the input materials including drug substances, excipients, in-process materials, while CQAs are for output materials, i.e., the product.

Implementing a risk assessment is vital to identify formulations, ingredients, or process parameters that can impact CQAs after the risk analysis appraises the impact of these parameters on the CQAs. Additionally, a qualitative or quantitative scale is used to rate the risk of each identified factor for the desired CQAs. For this reason, a risk assessment scale has to be established based on the severity and dubiety of the impact on efficacy and safety. Effect analysis and the Failer mode can be used to identify CQAs. After the risk evaluation process a few of these parameters become potentially critical for the CMAs, which must have certain properties and must be selected within a reasonable range to guarantee the CQAs of the final product [25,26].

## 3. Development of Liposomes Using QbD

### 3.1. QbD in Liposomal Formulation

The quality of liposomal pharmaceutical products is affected by their contents, preparation, properties and manufacturing key variables [12]. Therefore, QbD involves designing the final liposomal products by optimizing input material and manufacturing processes to acquire a pharmaceutical product with superior quality [27]. Moreover, QbD classifies and translates the critical parameters and key variables to produce a high-quality drug product with the most desired characteristics [28]. Indeed, several liposomal products have been developed using QbD approach, as summarized in Table 1.

To certify the desired quality of the final pharmaceutical product, a quality target product profile (QTPP) should be established [27]. QTPP is usually performed based on the available scientific data and proper in vivo significance [25]. To identify the QTPP and the process key parameters that can influence the liposomal product’s quality attributes (CQAs), the following principal CQAs generally should be recognized/optimized: average particle size, particle size distribution, zeta potential, drug content, in vivo stability and drug release [25,38].

Although there are many benefits of applying QbD to liposomal-based products, there are many challenges that limit the application of QbD liposomal-based product development. Benefits and challenges are summarized in Table 2.

### 3.2. QbD Process Key Parameters for Liposomal Products

#### 3.2.1. Lipid Type and Content

The integrity and stability of liposomes mainly rely on the lipid type. Lipids with unsaturated fatty acids are susceptible to degradation by hydrolysis or oxidation, while saturated fatty acids are more stable and have higher transition temperature (T_m_) [39]. Moreover, liposomes fluidity, permeability and surface charge also count on the lipid type and the liposomal lipidic composition [40]. For example, cholesterol typically increases liposome stability but should be optimized and not exceed 50% [41]. Generally, the carbon chain length of the formulated lipids may affect the drug encapsulation efficiency of both hydrophilic and hydrophobic drugs [40]. For example, a large aqueous core can be obtained using short fatty acid lipids that can enable a high internal volume for hydrophilic drugs. In contrast, long carbon chain lipids are more suitable to encapsulate the hydrophobic drugs within the hydrophobic lipid bilayer [42,43]. Furthermore, the loaded material has a great influence on the morphological features of the particles. The concentration of nucleic acids impacts the change from a multilamellar to an electron-dense morphology in lipidic-based particles [44].

Since 1978, liposomes have been used for the selective insertion of exogenous RNA into cells [45]. Many liposomes have been optimized and fabricated to encapsulate nucleic acids with low toxicity and high efficiency [46]. However, ionized lipids, especially cationic lipids, are still the most used for this purpose [47,48]. Unfortunately, cationic lipids produce many changes in the cell and proteins, such as cell shrinking, reduction in mitoses and changes in protein kinase C and cytoplasm vacuoles [49,50]. On the other hand, compared with viral vectors for gene delivery into cells, cationic lipids are easy to fabricate, simple and possess lower immunogenicity [51]

Both hydrophobic and hydrophilic parts of the cationic lipids have a toxic effect, especially if they contain a quaternary amine that acts as a protein kinase C inhibitor [52]. A new approach to decrease the effect of the positive charge was proposed to spread the charge by delocalizing it into a heterocyclic ring imidazolium [53] and a pyridinium [54]. Chang et. al., developed cationic lipids with a cyclen headgroup and revealed that this novel lipid is safer and possesses lower cytotoxicity than the commonly used lipid to deliver gene therapy [55].

#### 3.2.2. Manufacturing Process

**Figure 3:**
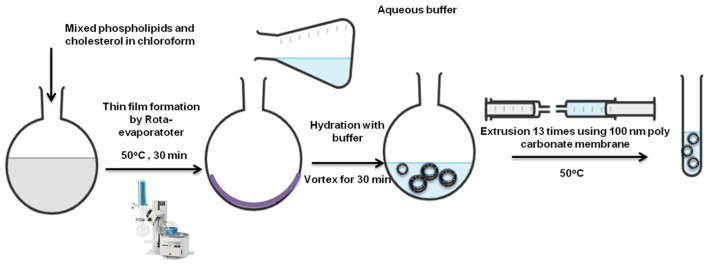
Liposomes preparation via thin-film hydration extrusion technique [57].

The most commonly used manufacturing process for liposome preparation is the thin-film hydration method (Figure 3) [56,57]. Other approaches such as reverse-phase evaporation, ethanol injection and emulsification have also been applied [58]. The thin-film hydration method produces multilamellar structure liposomes with an average diameter in micrometers [42]. Thus, resizing liposomes to less than 200 nm is required to improve the surface area to volume ratio for superior encapsulation and drug loading efficiency. Improving the size distribution of the prepared liposomes, extrusion, sonication (probe or bath) and freeze–thaw cycling have been used for liposomes size reduction [59].

Various parameters can be optimized to achieve a uniform multilamellar thin film followed by proper size reduction [43]. Rota-evaporator temperature, rotation speed and gradual pressure reduction, in addition to membrane pore size, can result in unilamellar, monodispersed liposomes with high encapsulation efficiency [33,60].

#### 3.2.3. Average Particle Size and Nanoparticles Distribution

Average particle size and nanoparticle distribution are considered the main CQAs for all nano-formulations [61]. These parameters play major roles in determining the nanoparticle in vivo distribution, drug loading ability, drug release and targeting capacity [62]. For better biodistribution, the ideal nanocarrier particle size should be in the range of 10 to 100 nm to avoid kidney elimination, escape the reticuloendothelial system (RES) and provide an effective enhanced permeability and retention (EPR) effect [63,64]. A small particle size means a high surface area to volume ratio. This leads to fast drug release due to more drugs close to the surface of the nanoparticles compared to larger ones [65]. However, it is important to keep in mind that for inhaled drug particles to be therapeutically useful, they should be smaller than 2 µm, which is most suitable for deposition in the alveolar [66]. Moreover, liposome delivery through the skin is dependent on size. Liposomes up to 600 nm penetrate through the skin easily, whereas liposomes larger than 1000 nm remain interiorized in the stratum corneum [67]. The polydispersity index (PDI) reflects the homogeneity and size distribution of the nano-dispersions. PDI values of less than 0.3 indicate homogeneous, stable and well-dispersed liposomes [68]. Generally, increasing lipids concentrations can lead to increased liposomal size and PDI values simultaneously [69].

#### 3.2.4. Zeta Potential (ZP)

ZP evaluates the nano-dispersion stability. Neutral nanoparticles have decreased stability and tend to aggregate [70]. A charge greater than +30 or less than −30 mV indicates good stability due to the high electrostatic repulsions [71]. The ZP of the nano-system affects their systemic circulation, interactions with body tissues and cell recognition. For example, the cellular uptake of cationic liposomes is higher compared than anionic liposomes due to the negatively charged cell membrane [72]. Moreover, charged liposomes can exhibit a high encapsulation efficiency for drugs with opposite charges [73]. In order to control the ZP values to achieve maximum stability, fatty acids and hydrophilic polymers of varying change can be incorporated into the liposome formulations [40].

#### 3.2.5. Drug Content

Liposomal drug content can be expressed in three ways: weight per volume (*w*/*v*); percentage encapsulation efficiency (EE%, weight of drug entrapped into the liposomes compared to the initial amount of drug used %); and drug loading (DL%, the amount of drug entrapped into the liposomes relative to the initial mass of the lipid used; drug-to-lipid ratio) [62,74]. Improved EE% preserves high concentrations of the precious pharmaceutical agent in liposomes and may reduce the manufacturing cost, thus resulting in enhanced pharmacokinetics and improved patient compliance [75].

Several parameters may influence the drug EE, such as the lipid-to-drug ratio, nature of phospholipids, cholesterol molar ratio and the manufacturing process parameters [76,77]. Increasing the lipid-to-drug ratio leads to an increase in the number of nano-vesicles that are able to entrap more hydrophilic drugs in their aqueous cores [78]. Cholesterol and unsaturated lipids create more pockets within the lipid bilayer, thereby entrapping more hydrophobic drugs [79,80]. Freeze–thaw resizing cycles have also been proven to enhance the EE [81]. Moreover, remote loading approaches into preformed liposomes have been able to raise the EE of ionizable drugs compared to conventional passive loading [82,83].

#### 3.2.6. In Vivo Stability

The hydrophobic/hydrophilic characteristics of the liposomes surface affect liposome interaction with blood components [84]. These interactions are responsible for the in vivo stability of liposomes. Liposomal in vivo stability causes prolonged drug release and enhanced drug localization in the targeted tissue [42]. For example, hydrophobic nanoparticles are easily cleared from blood circulation due to their high ability to bind blood proteins [38]. Moreover, stealth liposomes, usually coated with hydrophilic polymers, show higher in vivo stability with prolonged circulation time that leads to improved therapeutic potential of the encapsulated drug [70].

#### 3.2.7. Drug Release Kinetics

The kinetics of releasing drugs from liposomes is a critical parameter for liposome formulation design and considered a key factor to accomplish optimal efficacy and to minimize drug toxicity [85]. The optimal therapeutic activity of the drug can be achieved when the whole drug delivery system enters the target cells via endocytosis or the drug is released at the proper rate at the site of action for enough time [86]. Furthermore, the liposomes surface can be functionalized with targeting ligands for active drug targeting [87]. These targeting ligands can selectively bind to certain receptors or biomarkers that are overexpressed on cancerous or diseased tissues. These ligands could be antibodies, peptides, oligonucleotides, small carbohydrates, or small organic molecules [88].

Triggered drug release from liposomes could be achieved by incorporating sensitive excipients within liposome structures [89]. These excipients produce a liposomal destabilizing effect upon exposure to specific stimuli, such as light, temperature, radiation or different pH [90,91].

### 3.3. Product and Process Design Space

For the effective implementation of QbD in liposomal formulation, QTPP should be first defined, then the formulae and manufacturing processes can be selected and designed to ensure achievement of the pre-defined QTPP. Identification of CQAs and CPPs is achieved by an experimental design that is capable of assessing their contribution to the CQAs [62].

DS is performed to assure a high-quality product through demonstrating a range of process and/or formulation parameters [62,75]. DS involves the product and process DS. The product DS is established with the products CQAs as scopes, while the process DS is presented as CQAs related to CPPs [92].

The DS for liposome preparation is established by understanding and controlling the formulations, materials and manufacturing variables. Alina et al. established a DS for lyophilized liposomes with the drug simvastatin [32]. Their DS approach was based on both formulation factors and CPPs. Their results showed that cholesterol molar ratio, the PEG proportion, the cryoprotectant to phospholipids amounts and the number of extrusion cycles were designated as the most significant factors for lyophilized liposome CQAs [32]. These parameters were proven to directly affect the QTPP, including proper particle size, high drug entrapment, proper lyophilization process and minimum changes in phospholipid transition temperature. This DS approach was validated and considered a valuable approach for designing stable high-quality lyophilized liposomes [32].

This DS methodology was also applied to the prednisolone-loaded long-circulating liposomes using the thin-film hydration-extrusion method. The selected formulation parameters were drug concentration and PEG ratio in the bilayer membrane, and the process parameters were the number of extrusion cycles, temperature and rotation speed [33]. The same DS strategy was used to encapsulate tenofovir into liposomes with high EE [62]. Pandey et al. established a DS for chitosan-coated nanoliposomes using the ethanol injection method as a function of drug and chitosan concentration, and the organic phase-to-aqueous phase ratio to achieve the best design, in terms of average particle size, EE and coating efficiency [60].

Several factors may affect CQAs in the DS strategy. For example, the co-encapsulation of two drugs in the same liposome expands the studied attributes that are related to both drugs which are usually independent of each other. These variations may not lead to enhanced product quality [37]. Moreover, liposome drying process parameters are considered major CQAs that should be involved in the DS process study to obtain long-term stable liposomes [93]. Drying steps, such as pre-freezing, lyophilization and/or spray drying or even the type and ratio of the used cryoprotectants should be managed to reach a high drug content after lyophilization, maintaining the same particle size and ZP with minimal moisture content [94]. For example, the DS for the freeze-drying process of pravastatin-loaded long-circulating liposomes was developed as a function of the freezing rate and the shelf temperature during the initial drying. The two processing factors were found to have a great influence on the product’s CQAs [34].

### 3.4. The Control Strategy

Although liposomes have been shown to have many advantages as a stable and effective drug delivery system, they present many challenges in analytical and bioanalytical characterization due to their distinctive preparation processes and complex physicochemical properties. According to the FDA guidelines, numerous critical quality attributes (CQAs) have been reported that need full characterization for liposome drug products (Table 3).

#### 3.4.1. Lipid Content Identification and Quantification

The quality of the ultimate product is affected by the source of lipids and also by the nature of the lipids: synthetic, semi-synthetic or natural. Phospholipids are the major lipid component of liposome formulations. These lipids can be identified by nuclear magnetic resonance (NMR). ^31^P-NMR can differentiate phospholipid types according to their unique ^31^P shifts [95]. ^1^H- and ^13^C-NMR can also be used to clarify the molecular chemical structures of alkyl chains and lipid polar head groups. NMR analysis usually requires expensive instruments [96]. Liquid chromatography (LC) coupled with mass spectrometry (MS) is widely used for lipid identification and profiling [136]. MS is a powerful tool to determine the molecular mass of lipids especially when soft ionization approaches such as electrospray ionization (ESI) MS are used [137]. Raman spectroscopy can be used to characterize the vibrational modes of the lipid carbon skeleton. They are characterized by the C-C backbone vibrations (1000−1150 cm^−1^) and C-H stretching (2800−2900 cm^−1^) [138].

Liquid chromatography techniques have been widely applied in quantitative lipid analysis [139]. First, liposomes should be disrupted using organic solvents followed by chromatographic separation; then, lipids can be sensed and quantified by different detectors, including diode array ultraviolet (UV), refractive index (RI) [97], evaporative light scattering detector (ELSD) [98] and charged aerosol detector (CAD) [99]. Singh et al. quantified the phospholipids and cholesterol from six different liposomal preparations using isocratic, reversed-phase liquid chromatography (RP-HPLC) with UV and ELSD detectors [100].

Gas chromatography (GC) has also been applied for lipid analysis [102]. Lipid fatty acids should be first converted into volatile methyl esters prior to GC analysis [140]. Recently, supercritical fluid chromatography (SFC) has also been used for lipid analysis [103,141].

Many colorimetric assays have been stated to evaluate phospholipids. A blue-color is produced when reacting phosphorus with molybdate. Diphenylhexatriene (DPH) is usually used to identify bilayer membranes. Moreover, DPH fluorescence-based detection has improved the phospholipid concentration detection limits [101]. Additionally, several commercial kits have been designed to quantify unsaturated phospholipids based on the sulfo-phospho-vanillin reaction [142] or based on enzymatic assay [143,144].

#### 3.4.2. Quantification of Drug Encapsulation

Liposomes provide lipid bilayers and an aqueous core to entrap hydrophobic and/or hydrophilic drugs, respectively. To evaluate the drug encapsulation, the unloaded drug is first removed from the nanocarriers through ultrafiltration, ultracentrifugation, dialysis or solid-phase extraction. The loaded or unloaded drug amount can then be quantified with respect to the total drug amount, yielding the percent drug encapsulation [99].

RP-HPLC has shown high efficiency for both the separation and quantification of free drugs and drug-loaded liposomes [104]. RP-HPLC connected to a UV-detector has been used for fast quantification of doxorubicin-loaded into Doxil^®^ with a linear correlation [105,106]. Capillary electrophoresis (CE) has also been used to separate loaded drugs into liposomes of different change [107]. Oxaliplatin-loaded, anionic PEGylated liposomes have been purified from unloaded oxaliplatin and calculated for EE using a CE-UV detector [108]. Moreover, cisplatin has also been analyzed from loaded liposomes using CE connected to inductively coupled plasma mass spectrometry (ICP-MS) [109]. Flow-based field-flow fractionation (FFF) has been developed to overcome the restrictions of traditional chromatography [110,111]. Size exclusion chromatography (SEC) has also been used to separate unloaded drugs from drug-loaded liposomes based on their size differences [112].

#### 3.4.3. Liposomes Size and Morphology Characterization

**Figure 4:**
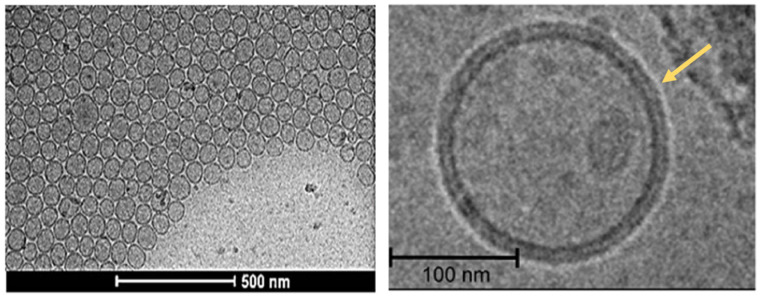
High-resolution Cryo-TEM images of liposomes [146].

Direct particle size and morphology can be evaluated by electron microscopy, such as scanning or transition electron microscopy (SEM and TEM, respectively) [113]. Cryogenic TEM (Cryo-TEM) does not require a drying process because it solidifies the aqueous sample by rapid freezing and thus drying-related artifacts are minimal. Cryo-TEM has been developed to provide high-resolution morphology and comprehensive structural information about the lipid layers and encapsulation mechanisms (Figure 4) [114,145]. SEM can penetrate the particle surfaces and is not commonly used for liposomal imaging due to the destructive manner of sample preparation. In addition, atomic fluoresce microscopy (AFM) has also been used to explore the three-dimensional structure of liposomes [115].

Liposome lamellarity can be evaluated by ^31^P-NMR [116]. Phospholipids in unilamellar liposomes can be characterized by a narrow-line spectrum, whereas multilamellar liposomes displayed wider peaks due to the restricted anisotropic molecular motions within multiple lipid layers [117].

Dynamic light scattering (DLS) has been applied to characterize nanoparticle size distribution. DLS has become the conventional strategy for the simple quantitative analysis of nanoparticle size distributions [118]. DLS measures time-dependent fluctuations in the scattered light from particles in Brownian motions. Variable sample parameters for DLS measurements include temperature, solvent viscosity and solvent refractive index, should all be pre-determined to precisely estimate the hydrodynamic particle size [119].

#### 3.4.4. Nanoparticle Surface Charge (Zeta Potential, ZP)

Liposomal surface charges are usually reflected by the polar head groups of the phospholipids, tertiary amines or negatively charged carboxylate functional groups. This factor is most often expressed by the ZP [120,121]. It is an important physicochemical property that is responsible for the strength of liposome interactions, adsorption and therefore nanoparticle stability. ZP can be determined from the electrophoretic mobility of particles measured by the phase analysis light scattering (PALS) or electrophoretic light scattering (ELS) technique [122]. Significant medium properties including the phase nature, refractive index, and viscosity, as well as temperature, all have to be pre-determined to obtain exact measurements. ZP values outside ±30 mV maintain sufficient stable nanosuspensions [123]. The surface potential of liposomes can also be determined by several techniques including fluorescent labeling [124], electron paramagnetic resonance [125] and the second harmonic generation from optical analyses [126].

#### 3.4.5. Physical and Chemical Stability

The physical and chemical stability of liposome formulations should be examined to meet the criteria for high product quality [147]. Spectroscopic methods and DLS measurements provide simple tests to measure liposome fusion and aggregation, respectively, while liposome disruption can be determined by chromatographic methods equipped with suitable detectors [42]. Liposomal fusion has been examined mainly using differential scanning calorimetry (DSC) and fluorescence-based lipid mixing assays [127]. Liposome aggregation can be envisaged by microscopic techniques and quantified by UV–Vis spectroscopy or DLS [128]. Lipid degradation rates can be affected by lipid composition, storage temperature, buffers and pH. The precursor lipid classes and their hydrolyzed derivatives can be separated and measured by several chromatographic approaches [129].

#### 3.4.6. In Vitro Drug Release

Several in vitro release testing methods to predict the in vivo behaviors of liposome formulations have been developed [130]. These methods can be classified into sampling and separate (SS), dialysis membrane (DM) and continuous flow (CF) [131,132]. The SS method involves incubating the samples in the release media, sampling and separating the released drug from integral liposomes, usually by stand-alone ultracentrifugation or filtration, followed by drug quantification [42,133]. Low-efficiency ultracentrifugation or filtration separation process for submicron nanoparticles has been observed upon using this method. DM is more common for studying the in vitro drug release of most nano-formulations. DM approaches mainly include dialysis sac (regular or tube dialysis) and reverse dialysis [134]. The dialysis sac keeps nano-formulations inside, attaining simultaneous release and separation, and then quantifying the released drug. Key factors for this approach include the type and cut-off of the dialysis membrane, volume ratios between the sample and release solvent, and mixing procedures [135].

#### 3.4.7. Liposomes Safety and Toxicity

The fact that liposomes are biocompatible, biodegradable and relatively easy to fabricate have led to an exponential increase in their use [148]. However, liposomes as a vehicle for drugs might be vulnerable to safety issues related to their lipid type, charge and concentrations. One of the most toxic effects of liposomes is the activation of the immune system of the patient that leads to drug sequestering in the mononuclear phagocytic system which might influence the function of the liver and spleen [149]. Therefore, strategies to improve the safety should be developed in the early stages of product design. Many strategies to improve drug safety and decrease the toxicity of the nanocarriers have been developed, such as increasing the encapsulation efficiency of drug into liposomes to decrease the lipid concentration needed to give the patient the recommended therapeutic dose [149]. The liposomes particle size, morphology, lipid content, charge, polydispersity and cholesterol content are key factors in toxicity. Consequently, precise design of all these factors will increase the loading capacity of liposomes and decrease the toxicity [150]. Recently approved were the PEGylated and surface-engineered liposomes having a lesser effect on the immune system. The combination of lipids with polymers should be designed and optimized. Therefore, the type of materials used for liposomal functionalization and their concentration should be minimized [148,151].

Finally, as the risk assessment is the backbone of the QbD process connecting all the key elements together, the liposomal biocompatibility and toxicity should be assessed using in vitro cell lines, ex vivo and in animals [152]. Many in vitro approaches have been used to test nanoparticle toxicity, including liposomes such as two-dimensional monolayer cell culture [153] and three-dimensional cell culture [154]. Additionally, ex vivo models are valuable tests systems in which slices of complete tissue can be used similar to organ slice cultures [155]. Finally, the most relevant evaluation is in vivo [156]. In conclusion, to minimize liposomal toxicity, it is important to start with the safety by design approach to ensure a low toxicity and voluble drug delivery system.

## 4. Conclusions and Future Perspectives

The application of QbD in pharmaceutical manufacturing has become an essential approach for the pharmaceutical industry to ensure the efficacy and safety of pharmaceutical products. The implementation of commercial nanomedicines as drug delivery systems to the site of action with limited systemic toxicities is an emerging concept that unfortunately, has not reached its full potential yet. Nano-pharmaceuticals are still in the initial stages of their development. Therefore, the implementation of QbD could create great value and benefits. Particularly, nano-pharmaceuticals is faced with many challenges related to structural stability and the lack of in-depth understanding of the manufacturing processes.

Liposomes are biocompatible and biodegradable drug delivery systems that have shown important successes in their clinical use. However, there are a lot of regulatory and technical challenges connected with the production and quality control strategies of liposomal products. There is a wide range of variability in liposomal preparations that include their morphology, size, fabricating materials, spatial configuration and manufacturing methods. Consequently, the application of a QbD approach in developing liposomes is critical and challenging compared to traditional dosage forms. Therefore, for the successful development of quality liposomal products, manufacturers need to consider employing QbD to identify and classify product attributes as well as material/process parameters with a deeper understanding of their complex interplay using proper experimental design and statistical analysis. QbD implementation is vital to ensure the final product attributes and the intended therapeutic and safety profiles.

## Figures and Tables

**Table 1:** Examples of pharmaceutical liposomes developed by QbD.

Drug	QTTPs	CMAs/CPPs/CQAs	Refs
Erlotinib	Dry powder, pulmonary route of administration, particle size, PDI, entrapment efficiency, content uniformity and assays	CQAs: particle size, PDI, entrapment efficiency. CMA: drug to lipid ratio CPPs: hydration time sonication time	[29]
Cefoperazone	Dry powder, pulmonary route of administration, particle size, PDI, entrapment efficiency.	CQAs: particle size, PDI, entrapment efficiency. CPPs: hydration time, sonication time	[30]
Lamotrigine	Nasal route, liquid formulation, one dose volume, dissolution profile/absorption time, vesicle size, pH	CQAs: vesicle/particle size (and size distribution), vesicle size: no aggregation, constant vesicle size.	[31]
Simvastatin		CQAs: size, liposomal SIM concentration, encapsulated solute retention, Tm change, water content.	[32]
Prednisolone	The vesicle size for tumor accumulation; PEGylation of the liposomes; an optimal cholesterol concentration for stability; a high concentration of incorporated drug	CPPs: rotation speed at the hydration of the lipid film and the extrusion temperature. CQAs: drug concentration, encapsulation efficiency and liposomal size.	[33]
Pravastatin	Systemic administration, accumulation at tumor site, improved stability, process efficiency.	CQAs: average particle size, encapsulated solute retention, zeta potential, residual moisture content, glass transition temperature, primary drying time, cake appearance.	[34]
Azacitidine	Particle size and % entrapment efficiency.	CPPs: lipid weight concentration (mg), cholesterol weight concentration (mg) and sonication time (min).	[35]
Salbutamol	Cholesterol concentration, phospholipid concentration, hydration time.	CPPs: drug to lipid ratio, drug entrapment efficiency, sonication time and hydration time. CQAs: vesicle size, zeta potential and drug encapsulation efficiency.	[36]
Doxorubicin-Curcumin	Decreasing doxorubicin (DOX) toxicity, enhancing curcumin (CUR) solubility, stability improvement.	CQAs: the size, surface charge, drug loading, EE and zeta potential. CPPs: buffer pH and temperature, phospholipid concentration, the phospholipids to cholesterol ratio and the extrusion temperature.	[37]

**Table 2:** Benefit and challenges of applying QbD in of liposomal-based products.

Benefits
Providing a better overall model for liposomal products with fewer problems in formulation and manufacturing Providing better understanding of the compatibility of ingredients in liposomes that affect the manufacturing process Enabling continuous improvements in liposomal formulation and manufacturing processes Avoiding regulatory problems and difficulties Understanding the associated risks to ensure consistent liposomal formulations Ensuring decisions that are based on optimized design rather than on empirical information Connecting liposomal formulations and manufacturing with clinical testing during design Accelerated FDA approval with less post approval modifications Minimizing post market changes and the total cost of liposomal formulation
Challenges
Increased research and development cost and time High initial cost of liposomal preparation, characterization and formulation Challenges in dosage form variability Regulatory and technical issues Increase in experimental runs due to increases in characterization variables of liposomes Difficulty in resolving the effect of confounders

**Table 3:** Critical quality attributes (CQAs) needed for full liposome drug product characterization.

CQAs	Measured Indicator(s)	Ref.
Lipid content and composition	- Total lipid assay - Composition determination	[95,96,97,98,99,100,101] [102,103]
Drug content	- Assay - Encapsulation efficiency	[99] [104,105,106] [107,108,109] [110,111] [112]
Liposome morphology, size and architecture	- Shape determination - Lamellarity - Average particle size and polydispersity indices	[113,114,115] [116,117] [118,119]
Liposome surface charge	- Zeta potential	[120,121,122,123,124,125,126]
Stability	- Liposomal fusion	[127] [122,128] [129]
	- Liposomes aggregation	
	- Lipid hydrolysis	
Drug release	- In vitro drug release	[130,131,132,133,134,135]

## Data Availability

Not applicable.
